# Supplementary material for: Causal interplay between lactose intolerance and gut microbiota: a combined bidirectional Mendelian randomization and in vivo validation study
Source: Front Nutr. 2026 Jun 1;13:1803337. doi: 10.3389/fnut.2026.1803337 (PMC13265576; doi:10.3389/fnut.2026.1803337)
Supplement: Supplementary file 1 [file Data_Sheet_1.zip › supplementary materials/Forward/forest plot/ebi-a-GCST90027792.finngen_R12_E4_LACTONAS.pdf]

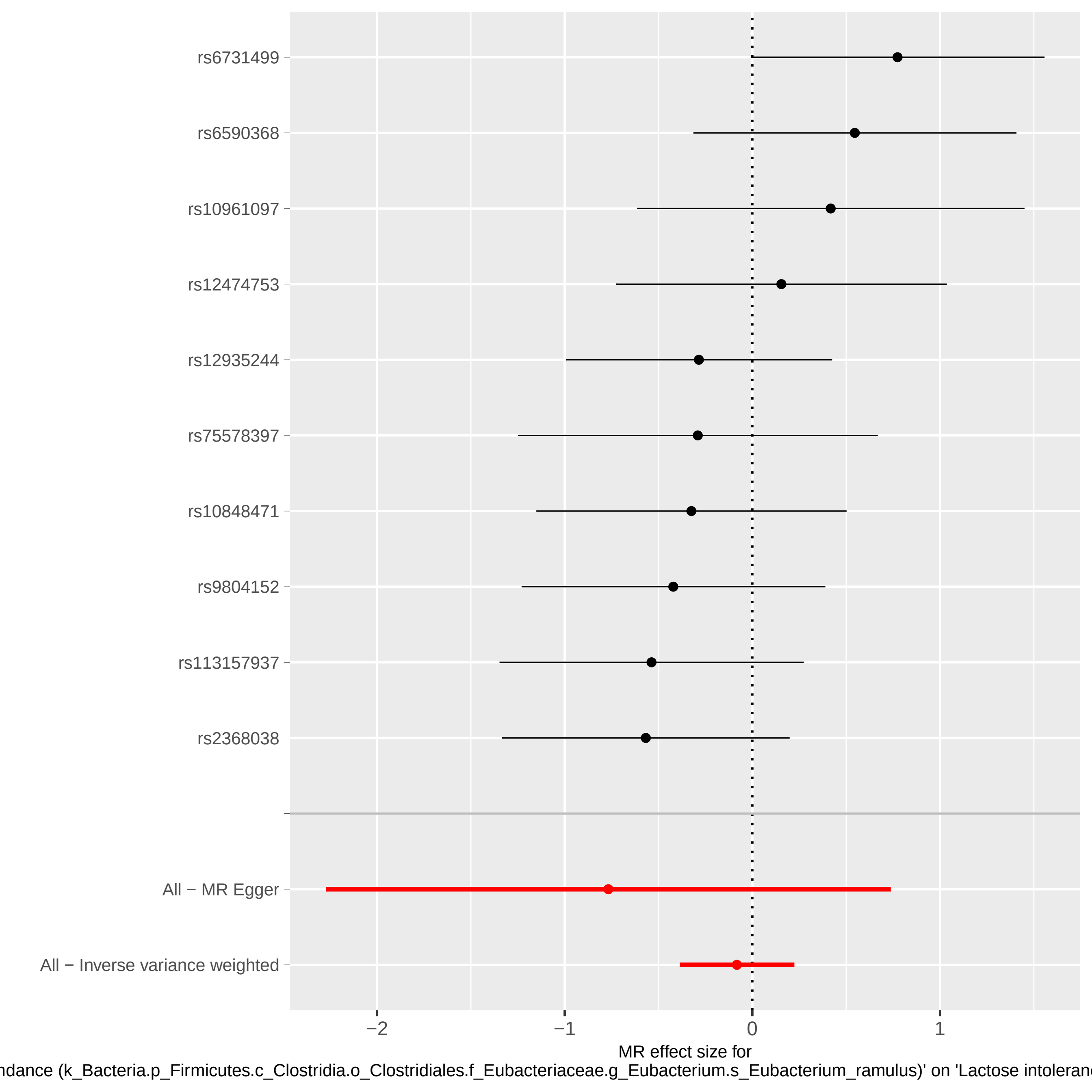
rs6731499
rs6590368
rs10961097
rs12474753
rs12935244
rs75578397
rs10848471
rs9804152
rs113157937
rs2368038
All − MR Egger
All − Inverse variance weighted
−2
−1
0
1
MR effect size for
ndance (k_Bacteria.p_Firmicutes.c_Clostridia.o_Clostridiales.f_Eubacteriaceae.g_Eubacterium.s_Eubacterium_ramulus)' on 'Lactose intoleran
